# Iron Deficiency and Nephrotoxic Heavy Metals: A Dangerous Interplay?

**DOI:** 10.3390/ijms24065315

**Published:** 2023-03-10

**Authors:** Pien Rawee, Daan Kremer, Ilja M. Nolte, Henri G. D. Leuvenink, Daan J. Touw, Martin H. De Borst, Stephan J. L. Bakker, Mark R. Hanudel, Michele F. Eisenga

**Affiliations:** 1 Department of Internal Medicine, Division of Nephrology, University of Groningen, University Medical Center Groningen, 9713 Groningen, The Netherlands; 2 Department of Epidemiology, University of Groningen, University Medical Center Groningen, 9713 Groningen, The Netherlands; 3 Department of Surgery, University of Groningen, University Medical Center Groningen, 9713 Groningen, The Netherlands; 4 Department of Clinical Pharmacy and Pharmacology, University of Groningen, University Medical Center Groningen, 9713 Groningen, The Netherlands; 5 Department of Pediatrics, David Geffen School of Medicine at UCLA, Los Angeles, CA 90095, USA

**Keywords:** iron deficiency, heavy metals, nephrotoxic, chronic kidney disease, metal transporters

## Abstract

Heavy metals are common in our environment, and all individuals are exposed to them to some extent. These toxic metals have several harmful effects on the body, including the kidney, which is a very sensitive organ. Indeed, heavy metal exposure has been linked to an increased risk of chronic kidney disease (CKD) and its progression, which may be explained by the well-established nephrotoxic effects of these metals. In this hypothesis and narrative literature review, we will shed light on the potential role that another highly common problem in patients with CKD, iron deficiency, may play in the damaging effects of heavy metal exposure in this patient group. Iron deficiency has previously been linked with an increased uptake of heavy metals in the intestine due to the upregulation of iron receptors that also take up other metals. Furthermore, recent research suggests a role of iron deficiency in the retention of heavy metals in the kidney. Therefore, we hypothesize that iron deficiency plays a crucial role in the damaging effects of heavy metal exposure in patients with CKD and that iron supplementation might be a strategy to combat these detrimental processes.

## 1. Introduction

The term heavy metals generally refers to metals that have a density five times greater than that of water [1]. Commonly known examples of such metals are cadmium, lead, and mercury. These metals have no known function in the human body and are toxic. Heavy metals are naturally occurring in our environment. Thus, complete avoidance of exposure is not feasible. Increased pollution of the environment with toxic metals has emerged due to the use of these metals in industry and agriculture [2]. However, in recent decades, heavy metal emission in Europe has started to decline [3]. In other parts of the world, such a trend is currently not seen and globally, metal emissions into the environment are still increasing [4,5]. Therefore, it remains an important public health problem since heavy metals can, even at low levels, have adverse effects on several aspects of human health, such as bone health, growth, and kidney function [6,7,8].

Humans are exposed to toxic heavy metals through several sources, including food, water, smoking, and air. Which route of exposure contributes most considerably to the concentrations of heavy metals within individuals depends on the region in which they reside and their habits. For example, in areas with high traffic density and substantial industrialization, air can be a significant contributor to metal exposure. Furthermore, for smokers, exposure to tobacco smoke significantly increases their metal levels. However, food and water contribute the most to metal exposure in the non-smoking and non-occupationally exposed general population [9]. Some examples of these routes of exposure are cadmium, found in food crops due to the use of cadmium-containing phosphate fertilizers, and lead in drinking water from lead pipes. Regarding dietary intake of metals, certain food groups contain especially high levels of heavy metals or contain moderate levels but are consumed in large quantities. For example, high seafood intake is linked to high urinary and blood levels of mercury [10]. Additionally, organ meats, such as liver and kidney, contain high cadmium levels, but these are generally only consumed in low quantities [11]. Furthermore, some vegetables grown in urban gardens have been reported to contain lead levels above health-based guidance levels [12]. The European Food Safety Authority reports that vegetables, grains, starchy roots, and tubers are the most significant contributors to dietary cadmium exposure [13]. Indeed, several studies have suggested that vegetarian and plant-based diets are associated with increased cadmium exposure [10,14,15]. Acute side-effects of high cadmium ingestion are gastrointestinal complaints, though a high-dose intake of cadmium through diet is uncommon [16]. Furthermore, cadmium exposure is linked to several cancer types, including lung, breast, prostate, nasopharynx pancreas, and kidney cancer, as well as impaired bone health [6,17].

The kidney is an organ prone to heavy metal-induced toxicity [18]. In this narrative review, we will discuss the consequences of heavy metal exposure to the kidney in the general population, patients with chronic kidney disease (CKD), and kidney transplant recipients (KTRs). Moreover, we will present our hypothesis that iron deficiency might play a crucial role in heavy metal-induced nephropathy.

## 2. Heavy Metals and the Kidney

Several heavy metals are known to be nephrotoxic. In this section, we first explain the association of several toxic metals with the risk of CKD in the general population and then we continue to explain the effects of heavy metals on individuals who already have CKD.

Multiple epidemiological studies have linked exposure to cadmium [19,20,21,22], lead [19,22], nickel [23,24], manganese [25], and the metalloid arsenic [19,22] to an increased risk of developing CKD in the general population. CKD is a clinical diagnosis often defined as an (estimated) glomerular filtration rate ((e)GFR) below 60 mL/min per 1.73 m^2^ or markers of kidney damage (e.g., proteinuria) for more than three months [26]. A meta-analysis reported a significantly increased risk of proteinuria for cadmium exposure (odds ratio (OR) = 1.25; 95% confidence interval (CI): 1.13–1.61) [19]. Another heavy metal well known to be nephrotoxic is lead. In the same meta-analysis, lead exposure was associated with increased risk of proteinuria (OR = 1.25; 95% CI: 1.04–1.49) and with decreased eGFR (<60 mL/min per 1.73 m^2^, OR = 1.12; 95% CI: 1.03–1.22) [19]. Similarly, arsenic was also associated with decreased eGFR (OR = 1.55; 95% CI: 1.05–2.28). Nevertheless, in this meta-analysis investigating the associations of cadmium, lead, and arsenic with CKD incidence, some heterogeneity was present. This suggests that inter-individual differences exist in the relationship between heavy metal exposure and risk of CKD [19]. The meta-analysis found no significant association between mercury, another known nephrotoxin [27], and CKD incidence. To date, studies investigating mercury exposure have reported that mercury is not linked with reduced estimated glomerular filtration rate (eGFR) [28,29,30,31], but whether an association with proteinuria exists has not been assessed yet. The research on the association of nickel with CKD incidence is limited and also heterogeneous. Two studies did identify an association between nickel and the development of CKD [23,24], whereas other studies did not [32,33]. Chronic exposure to nickel has been linked to tubular dysfunction in humans [34]. Furthermore, increased levels of manganese, an essential metal, were found in patients with CKD [25]. Interestingly, manganese has been found to reduce cadmium-induced toxicity in distal and proximal convoluted tubule cells [35], possibly because manganese and cadmium compete for the same transporters [36]. Finally, increased (but still in a relatively low range) levels of cobalt, another essential element, has been linked to decreased kidney function in the U.S. population when combined exposure with lead was present [37]. More large-scale prospective studies need to be performed to further delineate the link between exposure to different heavy metals and the risk of developing CKD in the general population.

In patients who already have CKD, the negative consequences of heavy metal exposure appear to be more pronounced. The increased perfusion/GFR of the remaining functional nephrons of individuals with early-stage CKD might cause increased exposure of the kidneys to toxic metals. This has also been implied in unilaterally nephrectomized rats exposed to cadmium [38]. With progression of CKD, the glomerular filtration rate decreases and, with that, the ability to eliminate heavy metals. The reduced elimination might contribute to cellular injury and enhance further disease progression [39,40]. Even relatively low cadmium levels in patients with CKD were associated with the progression to end-stage renal disease [41,42]. Furthermore, our group has found in KTRs that exposure to relatively low concentrations of cadmium and lead was associated with a substantially increased risk of developing graft failure [43,44]. KTRs can be particularly susceptible to toxic agents due to concomitant diseases (e.g., diabetes and cardiovascular disease), reduced kidney function, and adherence to immunosuppressive therapy. Since patients with CKD and KTRs appear to be particularly vulnerable to heavy metal toxicity, it is important to reduce exposure in these patient groups.

## 3. Handling of Heavy Metals by the Kidney

Heavy metals are filtered by the glomerulus and subsequently reabsorbed in the tubules of the kidney, mainly in the proximal renal tubules. These toxic metals can remain in the kidneys for a prolonged period. Cadmium, for example, has an estimated half-life of 45 years in the kidneys [45]. A clinical image that is often linked to heavy metal intoxication is Fanconi syndrome, characterized by generalized proximal tubular dysfunction. Among others, cadmium, lead, and mercury can induce Fanconi syndrome [46].

The mechanisms by which heavy metals are reabsorbed in the tubules vary across the different heavy metals. In Table 1, we provide an overview of the primary locations of accumulation and the most important transporters thought to be involved in renal handling of the different heavy metals.

Divalent metal transporter 1 (DMT1), a major iron transporter, has been suggested to transport a wide range of other divalent ions, including (in order of transport affinity) manganese (Mn^2+^), cadmium (Cd^2+^), iron (Fe^2+^), lead (Pb^2+^), cobalt (Co^2+^), nickel (Ni^2+^), and zinc (Zn^2+^) [47]. A later study has, however, indicated that only manganese, cadmium, iron, and cobalt are effectively transported by DMT1 [48,49]. DMT1 is widely expressed throughout the body [50], including in the intestines (where it plays a crucial role in iron absorption) and the kidney. DMT1 has been found, both apically and intracellularly, in the proximal and distal tubules [51]. Knockdown of DMT1 by small interfering RNA transfection in proximal tubule cells significantly reduced the amount of cadmium and manganese (both have relatively high affinity for DMT1 [49]) in the cells [52]. Thus, it seems that DMT1 plays a role in renal reabsorption of cadmium and manganese, but whether this transporter is also responsible for reabsorption of other divalent metal ions (that have lower affinity for this receptor) remains to be elucidated.

It has been suggested that zinc transporters ZIP8 and ZIP14, two other divalent metal transporters, are involved in the reabsorption of metal ions in the kidney. ZIP8 and ZIP14 are present at several places in the body, with the most abundant expression in the liver and lung, respectively. ZIP8 and ZIP14 also occur in the duodenum and the kidney [51,53,54]. These receptors can transport iron and have been suggested to do so in the kidney [55]. The role of ZIP8 and ZIP14 in intestinal iron absorption is less critical than the role of DMT1, which is necessary for sufficient intestinal iron absorption [56]. It has been suggested that ZIP8 and ZIP14 also transport cadmium and manganese [57,58]. Additionally, ZIP8 appears to transport cobalt [59]. Similar to DMT1, it is found that knockdown of ZIP8 and ZIP14 by small interfering RNA transfection in proximal tubule cells resulted in a significant reduction of cadmium and manganese in these kidney cells [52]. Less is known about lead uptake by these receptors, but it has been suggested that ZIP8 can transport lead, as lead reduces zinc uptake by the ZIP8 transporter [60]. Lastly, in mice with mutant ZIP8, cobalt levels in the kidney were reduced [61], suggesting a role for ZIP8 in cobalt reabsorption in the kidney.

Other important transporters that have been suggested to be involved in the renal handling of heavy metals include calcium channels for the reabsorption of lead and nickel [62,63,64]. Additionally, mercury has been proposed to be taken up from the peritubular blood into tubular epithelial cells by the organic anion transporter 1 (OAT1) on the basolateral side of the proximal renal tubule [65]. Knockout of this receptor in rats protected against mercury-induced kidney injury [66].

Importantly, all receptors described above transport divalent metal ions, but a large proportion of circulating metal ions is actually bound to proteins. For example, metallothionein (a low molecular weight protein) can bind several heavy metals, including cadmium [67]. Metallothionein binding can protect against metal toxicity, and heavy metal exposure induces the synthesis of this protein [67]. Furthermore, albumin has been shown to bind cadmium [68], nickel, and cobalt [69]. These proteins can be endocytosed in the proximal tubules by megalin–cubulin receptor complexes [70]. Megalin is a transmembrane receptor protein localized in the proximal tubule that operates in combination with the receptor protein cubulin and the transmembrane protein amionless. Metallothionein has a high affinity to cadmium. Previously, it was believed that cadmium was mainly reabsorbed in a complex with metallothionein by megalin–cubulin. However, as Fels et al. rightfully stated, the amounts injected in mice to study the reabsorption of cadmium were 2000-fold larger than physiological concentrations [68]. The affinity of megalin–cubulin for metallothionein is lower than the metallothionein levels measured in the ultrafiltrate, making it unlikely that metallothionein is taken up by megalin–cubulin in the proximal tubule to a large extent [68]. β2-microglobulin and albumin can also bind cadmium (although with lower affinity than metallothionein) and have a higher affinity for the megalin–cubulin complex than metallothionein. These protein complexes have been shown to cause toxicity in proximal tubular cells [68]. Lastly, it has been suggested that the lipocalin-2 receptor (located in the distal tubule) can also take up cadmium–protein complexes [71].

**Table 1:** An overview of divalent metal ions linked to nephrotoxicity.

Metal (Most Common Oxidation State)	Primary Locations of Accumulation	Receptors Suggested to Be Involved in Renal Accumulation
Cadmium (Cd^2+^)	Kidneys and liver [72]	Cd^2+^: DMT1 [52,73], ZIP8 ^1^ [52,74,75], ZIP14 [52] Cd^2+^- protein complexes: megalin–cubulin, lipocalin 2/NGAL/24p3 receptor [68,71,76]
Lead (Pb^2+^)	Bones [77]	Pb^2+^: Ca^2+^ channels [62]
Nickel (Ni^2+^)	Respiratory tract [78,79]	Ni^2+^: Ca^2+^ channels [64]
Manganese (Mn^2+^, Mn^4+^, and Mn^7+^)	Brain [80]	Mn^2+^: DMT1 [52], ZIP8 [36,52,81], ZIP14 [52]
Cobalt (Co^2+^ and Co^3+^)	Kidneys and liver [82]	Co^2+^: ZIP8 [61]
Mercury (Hg^+^ and Hg^2+^)	Kidneys [27]	Hg^2+^: OAT1 (basolateral) [65,66]

^1^ ZIP8 knockout in mice actually increased kidney cadmium levels, which was hypothesized to occur due to less storage of cadmium in the liver and, thus, more exposure to the kidney [83].

## 4. Hepatic Transporters and Heavy Metal Accumulation in the Kidney

Specifically for cadmium, it is known that after enteral absorption, it is first stored in the liver, where it is bound to metallothionein and then slowly released into the blood [76]. An interesting study with ZIP14 knockout mice suggested that this hepatic metal transporter might influence the levels of heavy metals that enter the circulation and reach the kidney. Remarkably, this study revealed that total body ZIP14 knockout in mice results in increased cadmium levels in the kidney [83]. This is surprising because ZIP14 has been proposed to play a role in the reabsorption of cadmium in the proximal tubule [52]. ZIP14 knockout did reduce hepatic cadmium levels. The researchers suggest that, because there is decreased uptake of cadmium in the liver, this results in higher blood cadmium levels and, thus, more cadmium exposure to the kidney [83].

Furthermore, a recent study investigated the effect of ZIP8 knockout on manganese levels in the kidney, liver, brain, heart, and small intestine [81]. Total body knockout of ZIP8 resulted in diminished manganese levels in the kidney. Interestingly, when only hepatic ZIP8 was knocked out, this resulted in a similar reduction in kidney manganese levels. Apparently, hepatic ZIP8 is a main regulator of manganese levels in other tissues [81]. Notably, in the ZIP8 knockout mice, hepatic manganese levels were also decreased. Thus, in the situation of ZIP8 knockout and manganese exposure, the reasoning mentioned above (that reduced liver uptake could cause increased circulating concentrations and, therefore, increased levels in the kidney) does not apply here. It is important to note that the hepatic expression of ZIP14 is approximately 10 times higher than the hepatic expression of ZIP8 [57].

The above-described renal metal transporters appear to play a role in heavy metal accumulation in the kidney. However, it can be speculated that when one of the individual renal transporters is absent or non-functional, this does not substantially impact renal accumulation, as metals have multiple pathways to enter kidney cells. Further research is warranted to understand the individual contributions of the several metal transporters in different tissues to the accumulation of heavy metals in the kidney.

## 5. Iron Deficiency in CKD

Iron deficiency is common among patients with CKD. In patients not on dialysis, prevalence rates of iron deficiency (defined as ferritin <100 mg/L and/or transferrin saturation (TSAT) < 20%) range from 15–73% [84,85]. In patients with CKD on hemodialysis, 16–36% have a TSAT < 20% [86,87]. Prevalence estimates of iron deficiency in KTRs range from 6–47% [88]. Iron deficiency among patients with CKD can be absolute or functional. Absolute iron deficiency implies having low total body iron stores, which can be caused by gastrointestinal bleeding, treatment with anticoagulants, antiplatelets, and proton pump inhibitors, and increased blood losses due to dialysis [89,90,91,92]. Additionally, patients can have functional iron deficiency, which refers to a situation where there is an impaired ability to utilize the body’s iron stores [55]. This is mainly due to the fact that patients with CKD have increased levels of hepcidin, a liver-derived 25-amino acid peptide that is a key regulator of iron homeostasis. Hepcidin regulates iron homeostasis by binding to ferroportin—the only known cellular iron exporter—on duodenal enterocytes and macrophages, causing its internalization and degradation. As such, hepcidin inhibits the release of iron from intracellular body stores to the circulation. Plasma hepcidin levels are increased when iron levels are high or as a result of inflammation (caused by the pro-inflammatory cytokine IL-6). In CKD patients, hepcidin levels are increased due to the low-grade pro-inflammatory state and reduced renal clearance of hepcidin [93,94]. As such, in patients with CKD, higher hepcidin levels [93,94] contribute to a state in which iron cannot be sufficiently mobilized or utilized. To further elaborate on our hypothesis that iron deficiency might play a crucial role in heavy metal accumulation, it is necessary to have a closer look at iron absorption in the gut and the handling of iron in the kidney.

## 6. Absorption of Iron in the Gut

**Figure 1:**
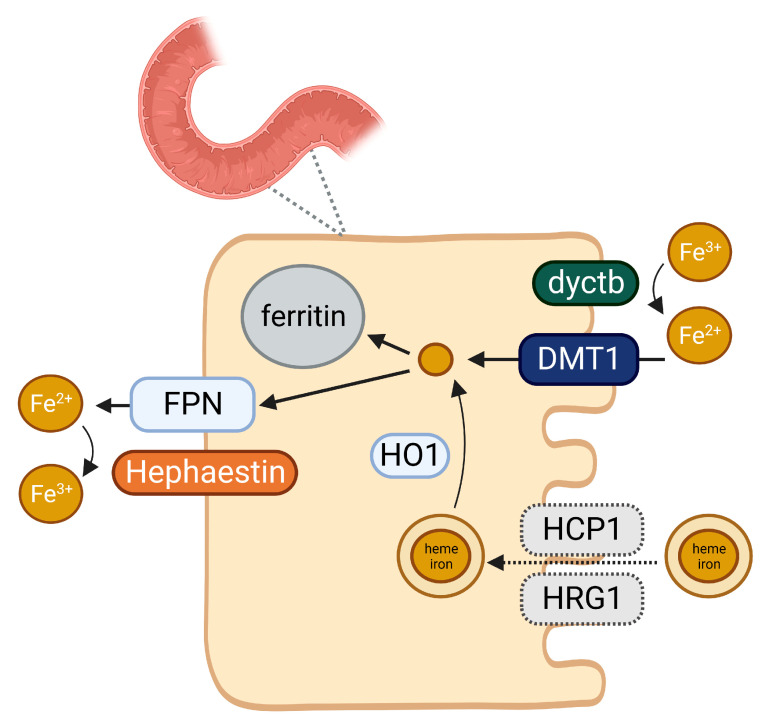
Overview of iron absorption in the gut. Dyctb: duodenal ferrireductase cytochrome B. DMT1: divalent metal transporter 1. HCP1: heme carrier protein 1. HRG-1: heme responsive gene 1. HO1: heme oxygenase-1. FPN: ferroportin. Created with BioRender.com.

Iron absorption in the gut is a sophisticated process (Figure 1). Dietary iron comprises two forms, namely, heme and non-heme iron. Heme iron is mainly present in animal-based foods, e.g., meat and fish, whereas non-heme iron is found in plant-based foods, e.g., vegetables and seaweed. Both heme and non-heme iron are absorbed at the apical brush border membrane of duodenal enterocytes. Iron absorption from heme iron (rate of around 25%) is more efficient than from non-heme iron (rate of around 5%) [95]. The absorption of non-heme iron is enhanced by ascorbic acid and inhibited by phytates (found in plant-based foods) and polyphenols (found in tea and coffee). Furthermore, calcium can inhibit the absorption of both heme and non-heme iron [96]. Iron must traverse the apical and basolateral membranes of duodenal enterocytes to reach the plasma. Ferric iron (Fe^3+^, the form in which most dietary iron presents itself) is enzymatically reduced to ferrous iron (Fe^2+^) by duodenal ferrireductase cytochrome B (dyctb). After reduction by dcytb, iron as Fe^2+^ is transported by DMT1 through the apical membrane of the duodenal enterocyte [95]. DMT1 thus forms the central mechanism by which iron is absorbed. Inside the enterocyte, iron can be stored as ferritin or be directly transferred to the circulation through ferroportin, the major iron exporter, located at the basolateral membrane. Ferrous iron transported through ferroportin is rapidly re-oxidized to ferric iron by hephaestin, the membrane-associated multicopper ferroxidase, or by ceruloplasmin, its soluble homolog. Heme iron absorption occurs through a different mechanism. It has been suggested that this mechanism involves heme carrier protein 1 (HCP1) and heme responsive gene 1 (HRG-1) protein [97]. Then, intracellular heme is degraded by heme oxygenase-1, which generates ferrous iron, whereafter, the same pathway is utilized as for non-heme iron [95].

## 7. Iron Handling in the Kidney

**Figure 2:**
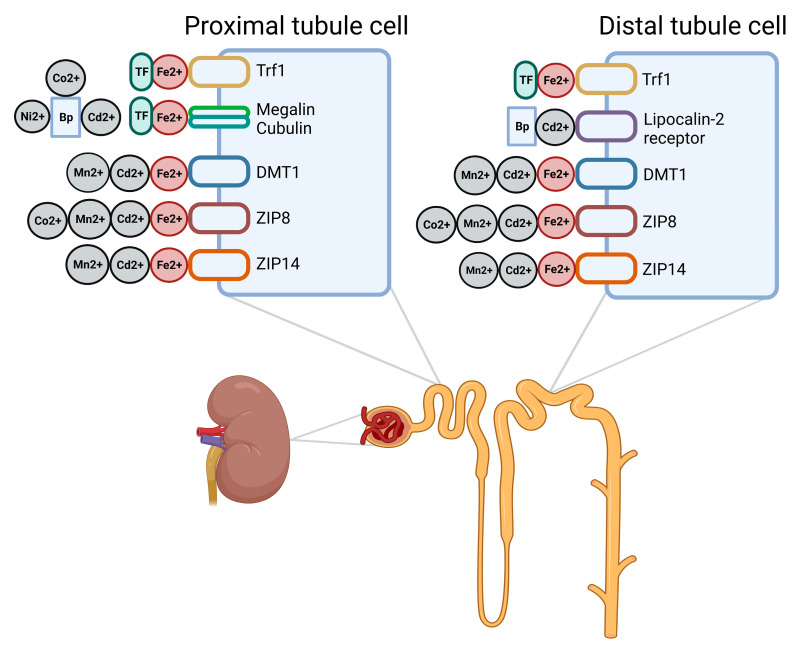
Overview of transporters suggested to be involved in the renal reabsorption of iron and other (toxic) metals. TF: transferrin. Trf1: transferrin receptor 1. Bp: binding protein, referring to different metal binding proteins, e.g., β2-microglobulin and albumin. DMT1: divalent metal transporter 1. Created with BioRender.com.

The kidney also plays a role in iron homeostasis. The most important receptors suggested to be involved are depicted in Figure 2. When circulating iron (bound to transferrin) reaches the kidney, it is filtered by the glomerulus. The rate of glomerular iron filtration has been estimated to be 10–30 µg per day [55]. Minimal levels of iron are found in the urine of healthy individuals, as the majority of filtered iron is reabsorbed. However, increased urinary iron levels are detected in individuals with tubular dysfunction [98,99], indicating that iron reabsorption is impaired in these individuals [51]. In the proximal tubules, the majority of iron is likely still bound to transferrin due to the neutral pH. Transferrin-bound iron from the lumen can be taken up by transferrin receptor 1 (Tfr1) and megalin–cubulin complexes. Trf1 expression is increased when iron levels are low (leading to more reabsorption of iron) and decreased when iron levels are high (leading to less reabsorption of iron). In contrast, the expression of the megalin–cubulin complexes in the kidney actually increases when iron levels are high [100]. Apparently, also when iron levels are high, there is reabsorption of transferrin-bound iron by megalin–cubulin complexes.

When pH drops below seven, iron dissociates from transferrin. There are several divalent metal transporters present on the apical side of proximal and distal renal tubular epithelial cells or intracellularly [51]. More specifically, the divalent metal transporters DMT1, ZIP8, and ZIP14 have been found in the proximal and distal tubules. Although these receptors are also found in the distal tubule, it is not expected that distal tubular cells play a significant role in the physiological iron handling of the kidney because the renal iron exporter ferroportin is only detected in proximal tubular cells [55]. Furthermore, as suggested by van Swelm et al., non-transferrin-bound iron in the proximal tubules is mainly transported by ZIP8 and ZIP14 and not DMT1, because the ZIP receptors work more effectively at the pH of the proximal tubules [55]. Knockout of ZIP8 in mice did not significantly reduce iron levels in the kidney [81], suggesting that ZIP14 or other receptors also substantially contribute to iron reabsorption.

## 8. Hypothesis: Iron Deficiency and Nephrotoxic Effects of Heavy Metals

**Figure 3:**
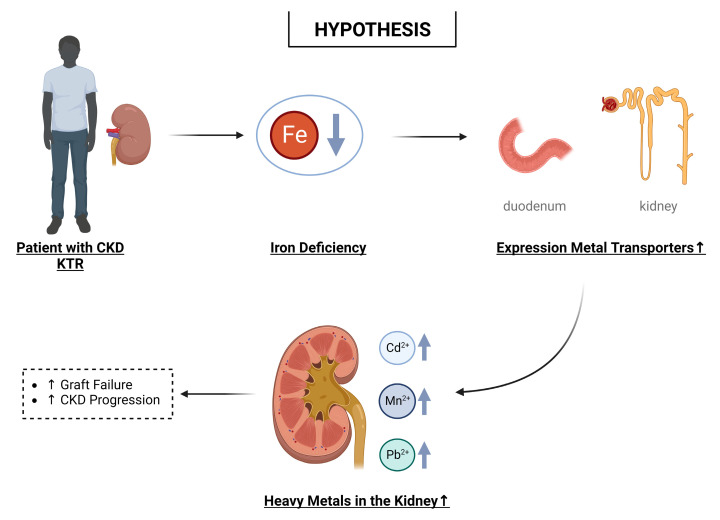
Overview of our hypothesis that iron deficiency, which is common in kidney (transplant) patients, plays a role in the nephrotoxic effects of heavy metal exposure. CKD: chronic kidney disease. KTR: kidney transplant recipient. Created with BioRender.com.

As highlighted in this review, a large overlap exists in the transporters by which iron and other heavy metals, e.g., cadmium [76] and manganese, are transported (see Figure 2). Since heavy metals such as cadmium have no known function in the human body, it can be assumed that the transporters of an essential metal, i.e., iron, are being utilized. Hence, our central hypothesis is that in the absence of iron, more divalent heavy metals are absorbed in the gut and retained in the kidney (see Figure 3). This interplay particularly jeopardizes patients with CKD and KTRs, who often present with iron deficiency (estimated prevalence in CKD patients; 15–73% [84] and in KTRs; 6–47% [88]). Furthermore, patients with CKD and KTRs appear to already experience the negative consequences of exposure to relatively low levels of heavy metals [41,42,43,44]. Possible explanations for this increased vulnerability are a reduced kidney function, concomitant diseases (e.g., diabetes and cardiovascular disease), and the adherence to immunosuppressive therapy (for KTRs). As every individual is exposed to heavy metals to some extent and low levels already appear harmful, metal nephrotoxicity might be a problem for a large percentage of CKD patients/KTRs and not only for those living in an area with high environmental levels or individuals with occupational exposure. The adverse effects of heavy metals might be worsened by the adoption of plant-based diets by patients with CKD. In general, diets rich in plant foods have positive health effects, and CKD patients are encouraged to adopt such a diet [101]. However, plant-based diets possibly increase the intake of toxic metals and might further increase the risk of iron deficiency in this patient group (i.e., potentially also resulting in increased uptake of these metals) [10,12,14,15].

Indeed, iron deficiency has been linked to increased plasma and tissue levels of cadmium in several human populations [102,103,104,105]. Furthermore, several studies have found a relationship between iron deficiency and increased levels of manganese and cobalt [106,107]. There is also some evidence for an association between iron deficiency and increased levels of lead [108,109,110]; however, this association appears to exist mainly in iron-deficient children. One recent study has linked lead exposure to iron deficiency in patients with CKD [111]. In animals, there have been several studies linking iron deficiency to increased levels of cadmium, manganese, and cobalt [112,113,114,115,116]. The main explanation provided for the association between iron deficiency and increased levels of heavy metals is that (1) there is upregulation of iron transporters that can also transport these toxic divalent metals and (2) there is decreased competition from iron to bind to these receptors.

Since people are often exposed to heavy metals through diet, intestinal iron transporters might play a particularly important role. It is known that the expression of DMT1, responsible for the uptake of iron in the duodenum, increases in the setting of iron deficiency [117,118]. Increased expression of DMT1 has been associated with higher cadmium levels in rats [115]. It can be speculated that increased expression of DMT1 induced by iron deficiency also increases the uptake of other divalent metal ions (besides cadmium) known to have moderate or high affinity for this receptor, including manganese and cobalt [49]. ZIP8 and ZIP14 are two other metal-ion transporters that are expressed in duodenum [53]. Both transporters can mediate the uptake of several metals, including iron, cadmium, manganese, and cobalt [52,54,57,58,59,119,120,121]. Although there have been mixed results regarding the effect of iron deficiency on ZIP8 and ZIP14 expression [54], it can be hypothesized that cadmium, manganese, or cobalt transport by these receptors increases when there is less competition from iron.

As mentioned above, the kidney is sensitive to heavy metal toxicity. Interestingly, many transporters thought to be involved in the reabsorption of heavy metals in the kidney also play a role in renal iron handling. In rat kidneys, it has been shown that there is increased expression of DMT1 in the presence of iron deficiency [122,123]. DMT1 in mouse proximal tubule cells can take up cadmium [52]. Given the known upregulation of DMT1 in an iron-deficient state, iron deficiency might also cause increased renal retention of other divalent ions that are effectively transported by this receptor (e.g., manganese and cobalt) [49]. Similar to the absorption of metal ions in the gut by ZIP8 and ZIP14, it can also be speculated that the renal iron transporters ZIP8 and ZIP14 increasingly reabsorb cadmium, manganese, and cobalt when there is less competition from iron.

It is relevant to mention that patients with CKD often have low-grade inflammation, which might impact the expression of metal transporters. For example, IL-6, which is increased in CKD patients, has been shown to upregulate ZIP14 expression in liver cells [124]. It would be interesting to study if similar upregulation of metal transporters occurs in CKD patients with functional iron deficiency.

## 9. Possible Implications

If our hypothesis is true, the possible interplay between iron deficiency and renal heavy metal accumulation might have multiple implications. Firstly, since even low levels of heavy metals can have detrimental effects on kidney (transplant) patients, it would be important to monitor heavy metal levels more frequently in this patient group and determine strategies to avoid exposure.

**Figure 4:**
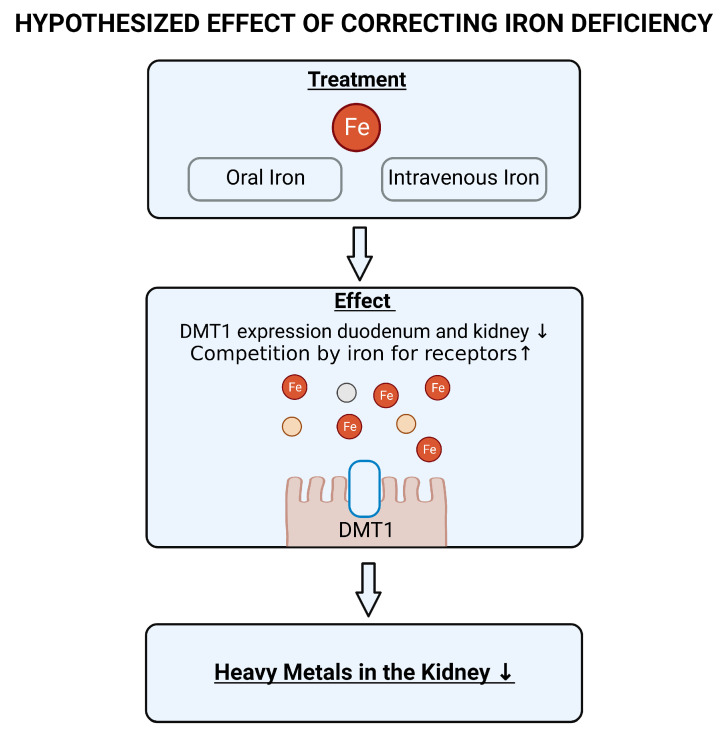
Hypothesized effect of correcting iron deficiency. DMT1: divalent metal transporter 1.

Furthermore, there are several potential therapeutic strategies against heavy metal induced toxicity. This includes the use of metal chelators such as ethylenediaminetetraacetic acid (EDTA). However, this chelator is non-specific, also binds iron (thus potentially further increasing iron deficiency) and has been reported to have no beneficial effect in individuals with renal dysfunction [125]. If iron deficiency indeed plays a clear role in toxic metal absorption and retention, then restoration of adequate iron stores might be a strategy to combat the increased retention of heavy metals in kidney patients (previously suggested for lead in [111]). Iron deficiency can be corrected by means of oral or intravenous supplementation. Advantages of oral iron are that it is accessible, inexpensive, and there is no association with severe side effects. However, gastrointestinal side effects are common and can negatively impact adherence to oral supplementation. Furthermore, impaired absorption of iron in the gut might reduce the efficacy of treatment with oral iron. Intravenous iron could thus be preferred over oral iron, but is occasionally associated with serious adverse events [126]. Current treatment guidelines for adult CKD patients recommend a trial with IV iron if an increase in hemoglobin without starting treatment with erythropoiesis-stimulating agents (medication that stimulates red blood cell production by the bone marrow) is desired, TSAT is ≤30%, and ferritin is ≤500 ng/mL. For non-dialysis-dependent CKD patients, a 1–3 month trial of oral iron therapy is recommended in this situation [126]. Although the importance of having adequate iron stores has already been clearly demonstrated in the setting of CKD [85,127,128], current practice demonstrates that many clinicians worldwide still do not adequately treat anemia and iron deficiency in CKD [129]. As iron deficiency is associated with increased expression of DMT1 in the gut and kidney, we expect that correcting iron deficiency will reduce the expression of this metal transporter (Figure 4). As a result, we expect there to be less absorption in the gut of metals that have affinity for DMT1 and less retention in the kidney. Furthermore, we expect that, in the presence of adequate iron stores, other metal transporters, such as ZIP8 and ZIP14, will be less likely to transport toxic metals due to the competition of iron. Thus, we expect that correction of iron deficiency will reduce the heavy metal burden of the kidneys.

## 10. Conclusions

In conclusion, CKD patients appear particularly vulnerable to the nephrotoxic effects of heavy metals. As a hypothesis, we propose that a highly common comorbidity in kidney (transplant) patients, i.e., iron deficiency, leads to increased uptake of heavy metals in the gut and increased retention in the kidney via upregulation of metal transporters. Since heavy metals occur in the environment, everyone will be exposed to them to some extent. Therefore, it is vital to further define the effects of heavy metals in this patient group and the role of iron deficiency therein. Supplementation with iron might represent a strategy to combat the potential detrimental effects of heavy metal toxicity in kidney (transplant) patients.

## Data Availability

No new data were created or analyzed in this study. Data sharing is not applicable to this article.
